# A Genome-Wide “Pleiotropy Scan” Does Not Identify New Susceptibility *Loci* for Estrogen Receptor Negative Breast Cancer

**DOI:** 10.1371/journal.pone.0085955

**Published:** 2014-02-11

**Authors:** Daniele Campa, Myrto Barrdahl, Konstantinos K. Tsilidis, Gianluca Severi, W. Ryan Diver, Afshan Siddiq, Stephen Chanock, Robert N. Hoover, Regina G. Ziegler, Christine D. Berg, Saundra S. Buys, Christopher A. Haiman, Brian E. Henderson, Fredrick R. Schumacher, Loïc Le Marchand, Dieter Flesch-Janys, Sara Lindström, David J. Hunter, Susan E. Hankinson, Walter C. Willett, Peter Kraft, David G. Cox, Kay-Tee Khaw, Anne Tjønneland, Laure Dossus, Dimitrios Trichopoulos, Salvatore Panico, Carla H. van Gils, Elisabete Weiderpass, Aurelio Barricarte, Malin Sund, Mia M. Gaudet, Graham Giles, Melissa Southey, Laura Baglietto, Jenny Chang-Claude, Rudolf Kaaks, Federico Canzian

**Affiliations:** 1 Division of Cancer Epidemiology, German Cancer Research Center (DKFZ), Heidelberg, Germany; 2 Cancer Epidemiology Unit, Nuffield Department of Clinical Medicine, University of Oxford, Oxford, United Kingdom; 3 Department of Hygiene and Epidemiology, University of Ioannina School of Medicine, Ioannina, Greece; 4 Cancer Epidemiology Centre, Cancer Council Victoria, Carlton South, Victoria, Australia; 5 Centre for Molecular, Environmental, Genetic and Analytic Epidemiology, School of Population Health, The University of Melbourne, Victoria, Australia; 6 Epidemiology Research Program, American Cancer Society, Atlanta, Georgia, United States of America; 7 Imperial College, London, United Kingdom; 8 Division of Cancer Epidemiology and Genetics, National Cancer Institute, Bethesda, Maryland, United States of America; 9 Division of Oncology, Huntsman Cancer Institute at the University of Utah School of Medicine, Salt Lake City, Utah, United States of America; 10 Department of Preventive Medicine, Keck School of Medicine, University of Southern California, Los Angeles, California, United States of America; 11 University of Hawaii Cancer Center, Honolulu, Hawaii, United States of America; 12 Department of Cancer Epidemiology/Clinical Cancer Registry, University Cancer Center Hamburg (UCCH), Germany; 13 Department of Medical Biometrics and Epidemiology, University Medical Center Hamburg-Eppendorf, Germany; 14 Harvard School of Public Health, Boston, Massachusetts, United States of America; 15 Channing Division of Network Medicine, Brigham and Women's Hospital and Harvard Medical School, Boston, Massachusetts, United States of America; 16 Division of Biostatistics and Epidemiology, University of Massachusetts, Amherst, Massachusetts, United States of America; 17 Université de Lyon, Université Lyon 1, Lyon, France; 18 Institut National de la Santé et de la Recherche Médicale U1052 Centre de Recherche en Cancérologie de Lyon, Lyon, France; 19 Centre national de la recherche scientifique UMR5286, Centre de Recherche en Cancérologie de Lyon, Lyon, France; 20 Centre Léon Bérard, Lyon, France; 21 Department of Public Health and Primary Care, School of Clinical Medicine, University of Cambridge, Cambridge, Cambridge, United Kingdom; 22 Danish Cancer Society Research Center, Copenhagen, Denmark; 23 Institut National de la Santé et de la Recherche Médicale, Centre for research in Epidemiology and Population Health, Nutrition, Hormones and Women's Health team, Villejuif, France; 24 Université Paris Sud, UMRS 1018, Villejuif, France; 25 Institut Gustave-Roussy, F-94805, Villejuif, France; 26 Bureau of Epidemiologic Research, Academy of Athens, Athens, Greece; 27 Hellenic Health Foundation, Athens, Greece; 28 Dipartimento di Medicina Clinica e Chirurgia, Federico II University, Naples, Italy; 29 Julius Center for Health Sciences and Primary Care, University Medical Center, Utrecht, The Netherlands; 30 Department of Community Medicine, Faculty of Health Sciences, University of Tromsø, The Arctic University of Norway, Tromsø, Norway; 31 Department of Research, Cancer Registry of Norway, Oslo, Norway; 32 Department of Medical Epidemiology and Biostatistics, Karolinska Institutet, Stockholm, Sweden; 33 Samfundet Folkhälsan, Helsinki, Finland; 34 Navarre Public Health Institute, Pamplona, Spain; 35 Consortium for Biomedical Research in Epidemiology and Public Health (CIBER Epidemiología y Salud Pública-CIBERESP), Madrid, Spain; 36 Department of Surgery, Umeå University Hospital, Umeå, Sweden; 37 Genomic Epidemiology Group, German Cancer Research Center, Heidelberg, Germany; IFOM, Fondazione Istituto FIRC di Oncologia Molecolare, Italy

## Abstract

Approximately 15–30% of all breast cancer tumors are estrogen receptor negative (ER−). Compared with ER-positive (ER+) disease they have an earlier age at onset and worse prognosis. Despite the vast number of risk variants identified for numerous cancer types, only seven loci have been unambiguously identified for ER-negative breast cancer. With the aim of identifying new susceptibility SNPs for this disease we performed a pleiotropic genome-wide association study (GWAS). We selected 3079 SNPs associated with a human complex trait or disease at genome-wide significance level (P<5×10^−8^) to perform a secondary analysis of an ER-negative GWAS from the National Cancer Institute's Breast and Prostate Cancer Cohort Consortium (BPC3), including 1998 cases and 2305 controls from prospective studies. We then tested the top ten associations (i.e. with the lowest P-values) using three additional populations with a total sample size of 3509 ER+ cases, 2543 ER− cases and 7031 healthy controls. None of the 3079 selected variants in the BPC3 ER-GWAS were significant at the adjusted threshold. 186 variants were associated with ER− breast cancer risk at a conventional threshold of P<0.05, with P-values ranging from 0.049 to 2.3×10^−4^. None of the variants reached statistical significance in the replication phase. In conclusion, this study did not identify any novel susceptibility loci for ER-breast cancer using a “pleiotropic approach”.

## Introduction

Estrogen receptor-negative (ER−) breast cancer (BC) comprises 15 to 30% of all breast tumours (depending on the population) and has an earlier age at onset and a worse prognosis compared with estrogen receptor-positive (ER+) disease. It is more common among women of African-American origin and it is also the breast cancer type associated with *BRCA1* mutations [1], [2]. Genome-wide association studies (GWAS) have identified thousands of common human genetic variants associated with risk of hundreds of quantitative traits and human diseases [3], [4]. Only seven susceptibility loci have been specifically identified for ER− BC [5]–[7]. In a GWAS, hundreds of thousands or even millions of polymorphisms are interrogated at the same time in a strictly agnostic way, i.e. ignoring any possible *a priori* knowledge of the SNPs tested. This model requires use of a stringent significance threshold (P<5×10^−8^) to correct for the numerous statistical tests performed and to avoid false positive findings. As a consequence, it is possible that variants with a truly positive but weak association are not detected and, therefore, not reported. A possible drawback of GWAS is that strict avoidance of false positives may lead to false negatives [8]. By running secondary analyses using a reduced number of SNPs defined by biological knowledge or hypothesis, the required threshold of significance may be lowered and the power to detect real associations of modest statistical effect may be increased.

A genetic mechanism termed pleiotropy, which is defined as one gene, or in this case allele, having an effect on multiple phenotypes [9] is an example for the selection of candidate SNPs for such secondary analysis. There are regions in the human genome, called Nexus, which have been associated with more than one distinct cancer type [10]. The most striking examples for cancer are: the 8q24 region, that harbors multiple *loci* associated with breast, colon, prostate, bladder and/or ovarian cancers, the *TERT* region, which has been associated with pancreatic, bladder, lung and prostate cancers, the p16 region on chromosome 9p21, and 6q25, and 11q13 associated, respectively, with non-Hodgkins lymphoma (NHL) and nasopharyngeal carcinoma and with bladder, breast and prostate cancer [10]. To the best of our knowledge a pleiotropic approach to identify novel cancer risk *SNPs* has been reported only once [11]. A pleiotropic GWAS performed to examine gene regions associated with pancreatic cancer, identified a region (*HNF1A*) previously associated with several diseases including Type-2 diabetes [12], [13].

We used a similar approach to search for new genetic variants associated with estrogen receptor negative breast cancer susceptibility. We selected all the SNPs that had been associated with a human disease trait or phenotype, at genome-wide level (P<5×10^−8^) and performed a secondary analysis on data from a GWAS study of ER− breast cancer by the National Cancer Institute's Breast and Prostate Cancer Cohort Consortium (BPC3) [7]. We then tested the top associations using three additional populations with a total sample size of 3509 ER+ cases, 2543 ER− cases and 7031 healthy controls.

## Materials and Methods

### Ethic statement

The Mammary Carcinoma Risk Factor Investigation (MARIE) study was approved by the ethics committees of the University of Heidelberg and the University of Hamburg. Written informed consent was obtained from all subjects.

For the BPC3 study written informed consent was obtained from all subjects and ethical approval was collected from the relevant institutional review boards from each cohort. The cohorts are: the European Prospective Investigation into Cancer and Nutrition (EPIC), the Melbourne Collaborative Cohort Study (MCCS), the Nurses' Health Study (NHS), the American Cancer Society Cancer Prevention Study II (CPS-II), the Prostate, Lung, Colorectal, Ovarian Cancer Screening Trial (PLCO), and the Multiethnic Cohort (MEC)

### Study populations

We performed the study in two phases: first we analysed data from the BPC3 ER− GWAS and second, for replication purposes, we used genotyping or existing data from selected breast cancer cases and controls collected by three different studies CPS-II, MCCS and the MARIE study. Individuals from CPS-II contributed cases and controls to both the initial GWAS and the replication phase, but there were no overlaps between sample sets used in the two phases of this study.

The BPC3 has been described extensively elsewhere [14]. It consists of cases and controls selected from large cohorts assembled in Europe, Australia and the United States that have both biological samples and extensive questionnaire information collected prospectively. Cases were women who were diagnosed with invasive BC after enrolment, the diagnosis was confirmed by tumor registries or by medical records. Controls were considered eligible if they were free of BC until the follow-up time for the matched case subject. Case and control subjects were matched for ethnicity and age and for some cohorts also for additional criteria, such as country of residence. Laboratory techniques and relevant QCs for the BPC3 ER− GWAS are extensively reported elsewhere [7]. Briefly, genotyping was performed at three centers (Imperial College London, UK, University of Southern California, USA, and the NCI Core Genotyping Facility, USA). Subjects from CPSII, EPIC, MEC, PLCO and PBCS were genotyped using the Illumina Human 660k-Quad SNP array (Illumina, San Diego, CA, USA), NHSI/NHSII and part of the PLCO study were genotyped previously using the Illumina Human 550 SNP array (Illumina, San Diego, CA, USA) [15]. For this study 1998 ER− invasive cancer cases and 2305 controls belonging to the BPC3 cohort were used.

The MARIE study population comprises BC patients who participated in a population-based case-control study conducted in two study regions in Germany (Hamburg and Rhine-Neckar-Karlsruhe). Cases were women diagnosed with histologically confirmed primary invasive or *in situ* breast tumor, aged 50 to 74 years, and residents of the study regions. Detailed information on tumor hormone receptor status was collected using clinical and pathology records. Controls were randomly selected from population registries and frequency-matched by year of birth and study region. The study has been described in more detail elsewhere [16]. For the present analyses, 2027 cases (370 ER−/1657 ER+) and 1778 controls were included.

### SNPs selection (phase one) and genotyping

The selection of the SNPs to be measured in phase one was done using the National Human Genome Research Institute's (NHGRI's) catalog of published GWA studies (http://www.genome.gov/gwastudies/) [4]. It contains summary information on polymorphic variants reported to be associated with a human disease, trait or phenotype in a GWA setting at the significance level of P<1.0×10^−5^. The data from the catalogue were downloaded in May 2012 and comprised 7986 SNPs. Approximately 60% (n = 5794) of the polymorphic variants reported in the catalogue had a P value higher than 5×10^−8^ and were, therefore, excluded from further analysis. Of the remaining 3192 SNPs, 1688 (58%) were genotyped in the BPC3 scan. PLINK [17] was used to identify highly correlated (r^2^>0.9 in Hapmap3 CEU) SNPs genotyped in the BPC3 GWAS for 452 variants (14.2% of the total selected SNPs). Data for 939 SNPs were imputed: 901 (28.3% of the total selected SNPs) from Hapmap 2 and 38 (1.1% of the total selected SNPs) from Hapmap3. The remaining 113 (3.6% of the total selected SNPs) variants were dropped from the analysis since no surrogate was found and it was not possible to impute data. Thus, data for 3079 out of 3192 catalogued SNPs (96.4%) were used for this study.

The 3079 remaining SNPs were looked up in the BPC3 GWAS ranking the P-value in decreasing order to check for their association. All already known breast cancer risk SNPs were excluded from the analysis.

### Replication (phase two) genotyping

In order to confirm the ten most significant findings we used additional BC cases and controls from three studies of women of Caucasian descent as a replication set: the CPS-II [18] consisting of 1530 estrogen receptor positive (ER+) cases, 53 ER− cases and 2395 healthy controls, the MCCS [19] with 322 ER+ cases, 122 ER− cases and 823 healthy controls, and the MAmmary carcinoma Risk factor InvEstigation (MARIE) [16] with 1657 ER+ cases, 370 ER− cases and 1778 healthy controls, for a total of 3684 cases and 4996 controls. Specifically rs498872, rs2000999, rs12150660, rs780094, rs11229030 and rs13397985 were replicated *in silico* for the MARIE, CPSII and MCCS studies. These six SNPs were genotyped as part of the iCOGS study using a custom Illumina array. In the original iCOGS publications SNPs with MAF <1%; call rate <95%; or call rate <99% and MAF <5% and all SNPs with genotype frequencies that departed from Hardy-Weinberg equilibrium at *P*<1×10^−6^ for controls or *P*<1×10^−12^ for cases were excluded [5], [20]. The remaining four SNPs rs8396, rs4788815, rs2571391, rs780092 were not present in the iCOGS array and were, therefore, genotyped *de novo* for the MARIE study by TaqMan. The mean genotyping success rate was 94.4% (88.2%–96.7%). The percentage of samples that was genotyped twice for quality assurance was 9.5%, the genotyping concordance was 99.99%. Departure from Hardy Weinberg equilibrium was tested for the ten SNPs for the respective control subjects from each study.

### Statistical analysis

Logistic regression adjusted for five principal components, age (at diagnosis for cases and at selection for controls) and cohort was used to generate ORs, 95% CIs, and P values for each of the 3079 SNPs selected from the BPC3 ER negative GWA data set and for the 10 SNPs in the replication phase. The replication was performed using ER− and ER+ breast cancer cases and the analysis was conducted using ER− alone and in combination with ER+. Considering the fact that several ER− SNPs are also associated with ER+ BC we included in the analysis ER+ and ER− cases and then analyzed overall BC risk (ER+ and ER−) and ER− specific (ER− alone) to increase our power to find a true association. We had more than 90% power to replicate any of the associations observed in the discovery phase if considering all BC cases, and over 50% (53%–72%) power if considering only ER− cases considering alpha of 0.05. Using a conservative Bonferroni correction, we considered a threshold of P-values<1.6×10^−5^ (0.05/3079) as statistically significant.

## Results

**Figure 1:**
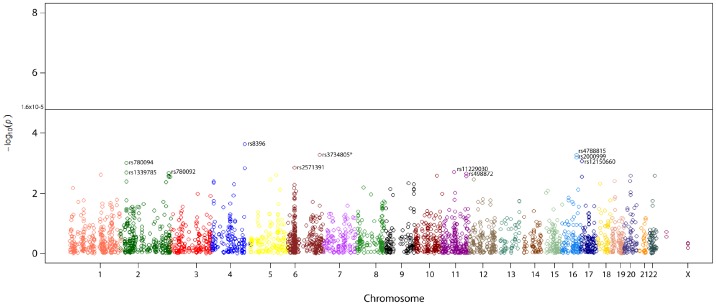
Manhattan Plot of all SNPs analyzed in phase one of the study.

None of the 3079 selected variants in the BPC3 ER-GWAS was significant at the adjusted threshold. 186 variants were associated with ER− breast cancer risk at a conventional threshold of P<0.05, with P values ranging from 0.049 to 2.3×10^−4^ (Figure 1). The strongest observed association was a decreased risk of ER− BC with rs8396 (OR_hetero_ :0.84; 95% CI 0.76–0.92 and OR_homo_0.71 (CI 95% 0.58–0.85)). We selected the most significant 10 SNPs (shown in table 1) and analyzed them using independent samples to determine whether they were genuinely associated with BC overall and for ER− breast cancer in particular. All the polymorphic variants were in Hardy-Weinberg equilibrium with the exception of rs12150660 in the CPSII and MARIE cohorts and rs13397985 in the CPSII cohort. Therefore, CPSII was not used as a replication set for rs12150660 and rs13397985 and MARIE was not used for rs12150660. In addition, one polymorphic variant rs8396 was not used in the analysis because it had a call rate lower than 95% (88.2%).

**Table 1:** The strongest associations between the pleiotropic SNPs and breast cancer risk.

SNP Name	study	ER status	OR^a^	95% CI^b^	P_trend^c^	study	ER status	OR	95%CI	P_trend
**rs2000999** [22]	BPC3	ER+/ER−	0.83	(0.75–0.93)	6.72E-04	BPC3	ER−	0.83	(0.75–0.93)	6.72E-04
	MCCS	ER+/ER−	1.05	(0.85–1.30)	6.30E-01	MCCS	ER−	1.14	(0.80–1.62)	4.80E-01
	CPS2	ER+/ER−	0.94	(0.85–1.04)	2.41E-01	CPS2	ER−	0.88	(0.53–1.46)	6.19E-01
	MARIE	ER+/ER−	1.02	(0.91–1.15)	7.26E-01	MARIE	ER−	0.99	(0.81–1.20)	8.85E-01
**rs12150660** [23]	BPC3	ER+/ER−	0.84	(0.76–0.93)	8.58E-04	BPC3	ER−	0.84	(0.76–0.93)	8.58E-04
	MCCS	ER+/ER−	1.00	(0.82–1.21)	9.70E-01	MCCS	ER−	0.79	(0.56–1.11)	1.70E-01
	CPS2	ER+/ER−	0.94	(0.84–1.05)	2.74E-01	CPS2	ER−	1.21	(0.74–1.98)	4.53E-01
	MARIE	ER+/ER−	1.16	(1.04–1.31)	1.07E-02	MARIE	ER−	1.18	(0.97–1.42)	9.87E-02
**rs13397985** [24]	BPC3	ER+/ER−	0.84	(0.76–0.94)	2.18E-03	BPC3	ER−	0.84	(0.76–0.94)	2.18E-03
	MCCS	ER+/ER−	1.09	(0.88–1.35)	4.20E-01	MCCS	ER−	0.95	(0.66–1.38)	8.00E-01
	CPS2	ER+/ER−	0.94	(0.82–1.08)	3.58E-01	CPS2	ER−	1.16	(0.65–2.07)	6.17E-01
	MARIE	ER+/ER−	1.11	(0.98–1.26)	8.65E-02	MARIE	ER−	1.16	(0.95–1.42)	1.46E-01
**rs780094** [25]	BPC3	ER+/ER−	0.87	(0.80–0.94)	9.97E-04	BPC3	ER−	0.87	(0.80–0.94)	9.97E-04
	MCCS	ER+/ER−	1.01	(0.86–1.19)	8.70E-01	MCCS	ER−	0.94	(0.72–1.24)	6.80E-01
	CPS2	ER+/ER−	0.96	(0.88–1.04)	3.00E-01	CPS2	ER−	0.98	(0.67–1.45)	9.29E-01
	MARIE	ER+/ER−	1.02	(0.92–1.12)	7.38E-01	MARIE	ER−	0.97	(0.83–1.14)	7.34E-01
**rs11229030** [26]	BPC3	ER+/ER−	0.87	(0.80–0.95)	1.95E-03	BPC3	ER−	0.87	(0.80–0.95)	1.95E-03
	MCCS	ER+/ER−	0.98	(0.83–1.15)	8.00E-01	MCCS	ER−	1.01	(0.77–1.33)	9.20E-01
	CPS2	ER+/ER−	1.02	(0.94–1.11)	6.60E-01	CPS2	ER−	1.31	(0.89–1.94)	1.72E-01
	MARIE	ER+/ER−	0.98	(0.89–1.08)	6.22E-01	MARIE	ER−	0.85	(0.72–1.00)	4.94E-02
**rs780092** [27]	BPC3	ER+/ER−	1.20	(1.07–1.34)	2.06E-03	BPC3	ER−	1.20	(1.07–1.34)	2.06E-03
	MARIE	ER+/ER−	1.03	(0.94–1.14)	4.96E-01	MARIE	ER−	0.90	(0.74–1.10)	3.17E-01
**rs4788815** [28]	BPC3	ER+/ER−	0.85	(0.78–0.93)	5.29E-04	BPC3	ER−	0.85	(0.78–0.93)	5.29E-04
	MARIE	ER+/ER−	0.99	(0.92–1.06)	6.94E-01	MARIE	ER−	1.14	(0.99–1.31)	7.50E-02
**rs2571391** [29]	BPC3	ER+/ER−	1.16	(1.06–1.27)	1.42E-03	BPC3	ER−	1.16	(1.06–1.27)	1.42E-03
	MARIE	ER+/ER−	1.03	(0.95–1.10)	4.82E-01	MARIE	ER−	0.91	(0.79–1.06)	2.25E-01
**rs498872** [30]	BPC3	ER+/ER−	1.15	(1.05–1.26)	2.21E-03	BPC3	ER−	1.15	(1.05–1.26)	2.21E-03
	MCCS	ER+/ER−	0.96	(0.80–1.15)	6.60E-01	MCCS	ER−	1.05	(0.78–1.43)	7.30E-01
	CPS2	ER+/ER−	0.92	(0.85–1.01)	7.10E-02	CPS2	ER−	0.96	(0.63–1.47)	8.50E-01
	MARIE	ER+/ER−	1.01	(0.91–1.12)	9.07E-01	MARIE	ER−	0.96	(0.80–1.14)	6.11E-01

a OR = Odds Ratio.

b 95% CI = 95% Confidence Intervals.

c All analysis were adjusted for age at diagnosis and in the BPC3 for cohort of provenience.

Only rs11229030, a variant originally found associated with risk of Crohn's disease, was nominally associated with a decreased risk of ER− BC (OR 0.85, CI 95% 0.75–1.00, P value = 0.049). The association was observed only for the MARIE study. The results of all the analyses are shown in table 1. Additional information on the original reports can be found at http://www.genome.gov/gwastudies/. We also performed meta-analysis between the various studies but the results were very heterogeneous, clearly suggesting a negative finding (Forest plots, heterogeneity P-values and I^2^ statistics are shown in figure S1).

## Discussion

Pleiotropy is a fairly common phenomenon that is defined as one gene or allelic variant having an effect on multiple phenotypes. In a recent paper based on data from the catalogue of published GWAS, Sivakumaran and colleagues have reported that 4.6% of the SNPs and 16.9% of the genes present in the catalogue are shown to have pleiotropic effects [9]. These percentages probably underestimate the real biological significance, since they have been obtained using a very conservative threshold, such as considering only the SNPs available in the catalogue and associated with a particular disease or trait at a genome wide level. Using data from GWAS meta-analyses, pleiotropy seems to play a much stronger role for specific diseases, for example Cotsapas and collaborators reported that 44% of the susceptibility loci for autoimmune diseases overlap [21]. In a two-staged analysis of 3509 ER+ cases, 2543 ER− and 7031 healthy controls, none of the SNPs showed a statistically significant association with breast cancer in the replication analysis. The strongest signal, in the replication analysis, was given by rs11229030 (a Crohn's disease susceptibility allele) that was associated with a decreased risk of ER− BC (P value = 0.049) only in the MARIE study, but not in CPS-II or MCCS suggesting that the association found is probably due to chance.

There are several possible reasons why our pleiotropic approach failed to identify new SNP associatated with ER− BC. First, ER− BC may be associated with uncommon biologic pathways that are not shared with many other diseases and, therefore, may not be influenced by pleiotropy. This is consistent with the fact that there are several SNPs which are specifically associated with ER− but not ER+. Alternatively, ER− BC may share genetic risk factors with other common disease traits and phenotypes, but not with those we included in our analysis. The pleiotropic approach we used is necessarily limited by the number of disease traits and phenotypes that have been examined with enough statistical power to identify GWAS hits. It is possible that disease traits and phenotypes with biologic pathways similar to ER− BC have not been examined adequately and are yet to be included in the NHGRI database.

We are aware of several limitations that this work might present: first, we were not able to include all the SNPs from the catalogue because 113 (3.6% of the total selected SNPs) variants were dropped from the analysis since no surrogate was obtained and it was not possible to impute data. Second, we replicated, as an exploratory analysis, only the 10 most significant SNP associations, thus we cannot exclude that a true positive association lies those SNPs that we did not attempt to replicate in the second phase, although due the complete lack of replication of the first ten SNPs this possibility seems unlikely. Third, we have included only the SNPs present in the GWAS catalogue, but we did not include other SNPs present in the regions. Since in pleiotropic regions the SNPs associated with different traits or diseases are not always the same, we can not exclude the possibility that we might have left out SNPs that are truly associated with ER− but that are not yet present in the GWAS catalogue. The other limitation is the sample size of the replication set which is quite large, considering the rarity of the disease, but might have been inadequate to detect weaker associations.

In conclusion, and given the limitations summarized, we did not identify any pleiotropic SNP associated with ER-breast cancer.

## Supporting Information

Figure S1 **Forest plots, I^2^ and heterogeneity P-values for the selected polymorphisms in the meta-analysis of the three studies.**
(DOC)
