# Supplementary material for: A Genome-Wide “Pleiotropy Scan” Does Not Identify New Susceptibility Loci for Estrogen Receptor Negative Breast Cancer
Source: PLoS One. 2014 Feb 11;9(2):e85955. doi: 10.1371/journal.pone.0085955 (PMC3921107; doi:10.1371/journal.pone.0085955)

**Supplementary Figure S1: Forest plots, I^2^ and heterogeneity Pvalues for the selected polymorphisms.**


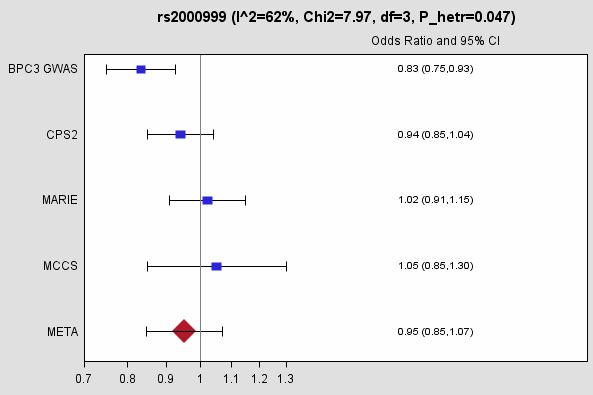


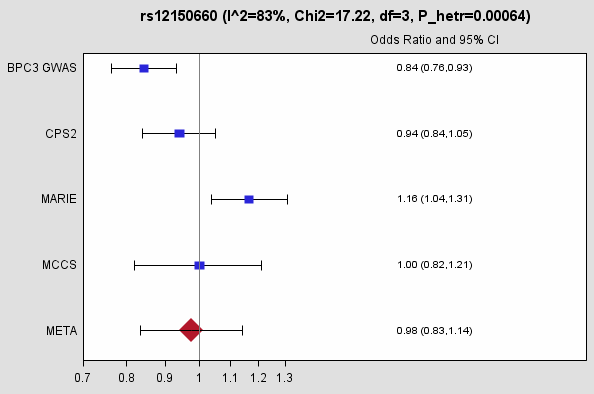


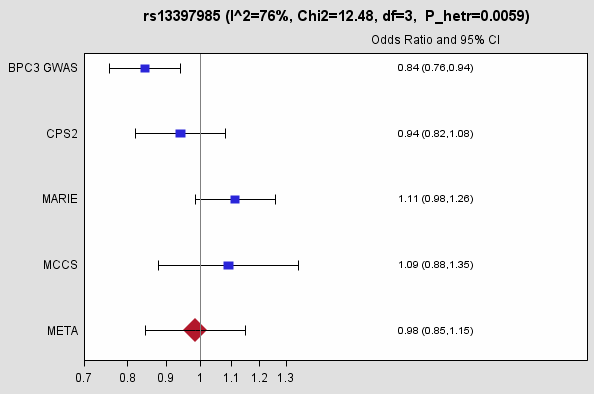


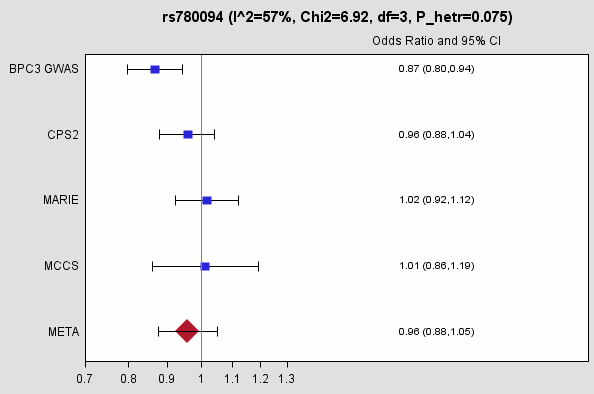


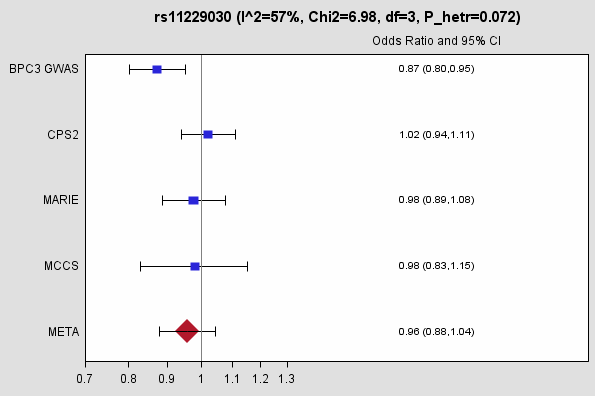


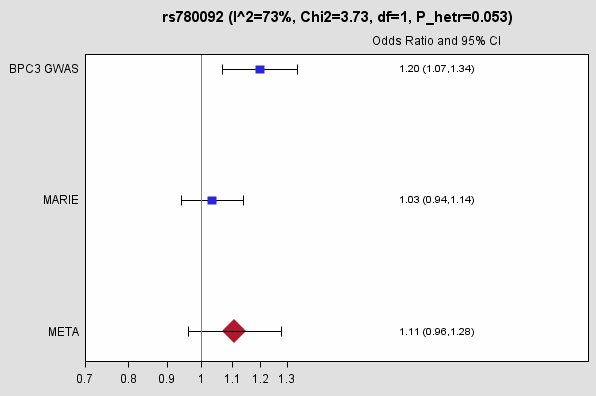


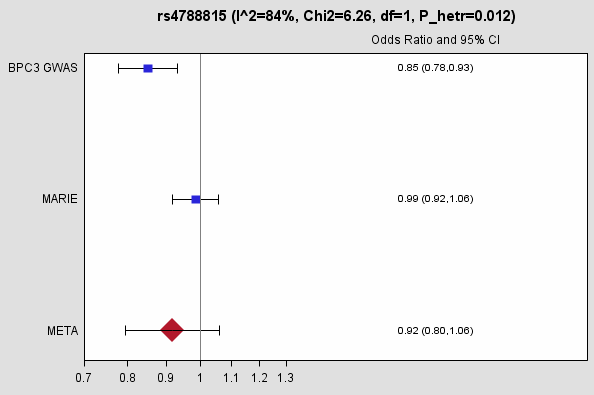


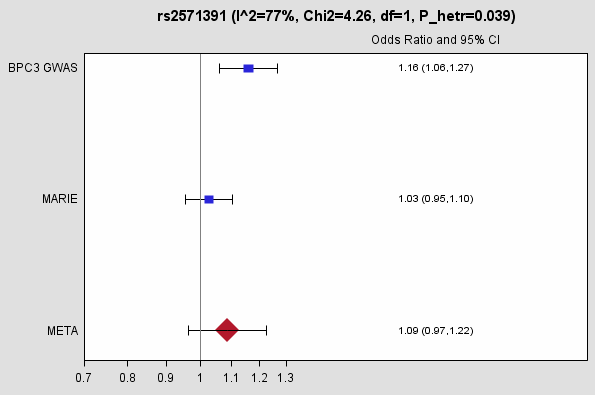


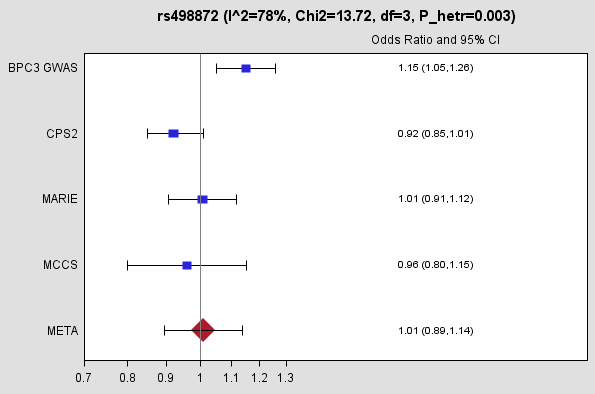

Supplement: Figure S1 — Forest plots, I2 and heterogeneity P-values for the selected polymorphisms in the meta-analysis of the three studies. (DOC) [file pone.0085955.s001.doc]
